# Robust classification using average correlations as features (ACF)

**DOI:** 10.1186/s12859-023-05224-0

**Published:** 2023-03-20

**Authors:** Yannis Schumann, Julia E. Neumann, Philipp Neumann

**Affiliations:** 1 Chair for High Performance Computing, Helmut-Schmidt University, Hamburg, Germany; 2 Center for Molecular Neurobiology Hamburg, University Medical Center Hamburg-Eppendorf, Hamburg, Germany; 3 Institute of Neuropathology, University Medical Center Hamburg-Eppendorf, Hamburg, Germany

**Keywords:** Classification, Machine learning, Correlation, Missing values, scRNA-seq

## Abstract

**Motivation:**

In single-cell transcriptomics and other omics technologies, large fractions of missing values commonly occur. Researchers often either consider only those features that were measured for each instance of their dataset, thereby accepting severe loss of information, or use imputation which can lead to erroneous results. Pairwise metrics allow for imputation-free classification with minimal loss of data.

**Results:**

Using pairwise correlations as metric, state-of-the-art approaches to classification would include the *K-nearest-neighbor*- (KNN) and *distribution-based-classification*-classifier. Our novel method, termed average correlations as features (ACF), significantly outperforms those approaches by training tunable machine learning models on inter-class and intra-class correlations. Our approach is characterized in simulation studies and its classification performance is demonstrated on real-world datasets from single-cell RNA sequencing and bottom-up proteomics. Furthermore, we demonstrate that variants of our method offer superior flexibility and performance over KNN classifiers and can be used in conjunction with other machine learning methods. In summary, ACF is a flexible method that enables missing value tolerant classification with minimal loss of data.

**Supplementary Information:**

The online version contains supplementary material available at 10.1186/s12859-023-05224-0.

## Introduction

### Background

With the increasing availability of high quality data across various disciplines, researchers commonly employ data mining techniques such as classification, clustering or regression to answer the research questions under consideration. Classification aims to assign new observations to one (or multiple) classes based on a set of training instances, e.g. assigning a diagnosis (sick/healthy) to a patient.

While powerful classifiers have been successfully implemented for commonly studied omic types, such as DNA-methylation [1] or the transcriptome [2], a widespread problem associated with many emerging omics technologies such as proteomics and single-cell RNA sequencing (scRNA-seq) is the strong prevalence of missing values, which hampers the direct applicability of most classification algorithms. The number of missing values is often additionally amplified by the integration of multiple individual datasets which is a common strategy to add statistical power to a study [3].

In order to overcome those hurdles, researchers often either delete all features with missing values (leading to significant loss of information) or use imputation methods that do not generalize well across datasets [4, 5] and that have been demonstrated to introduce false positives and irreproducible differential expression in certain cases [6].

In this paper, we present an approach that relies on pairwise correlations to train tunable machine learning models in a modular fashion. We make use of inter- and intra-class correlations and respectively pairwise deletions [7], resulting in minimal data loss and independence of potentially error-prone imputation.

### Related work

Multiple mechanisms contribute to the presence of missing data in omics datasets, such as for instance biologic differences between the samples, technical reasons (e.g. detection thresholds) or limitations of the bioinformatics pipeline (e.g. misidentification of peptides in mass spectrometric data). Based on such mechanisms, Rubin [8] introduced the established discrimination between different types of missing values into MCAR (missing completely at random), MAR (missing at random) and MNAR (missing not at random).

As elaborated by Emmanuel et al. [7], strategies to handle these missing values can be broadly divided into deletion and imputation. The latter uses the measured data to predict and replace the missing values. Extensive studies have been conducted to evaluate the strengths and weaknesses of various imputation methods on different omics types [6, 9, 10]. Lazar et al. [4] conclude that the involved missing value mechanism impacts the performance of imputation on label-free quantitative proteomics data and advocated the development of hybrid strategies that consider the coexistence of different types of missing values. Lately, tools have been developed to select suitable imputation methods in a data driven fashion to tailor the type of imputation to the dataset under consideration [5]. Most recently, Linderman et al. published the ALRA-algorithm, which discriminates between biological and technical zeros for scRNA-seq data and only imputes the latter [11].

Deletion-based approaches can be divided into listwise and pairwise deletion (cf. [7]). Listwise deletion refers to deleting all features that contain missing values whereas pairwise deletion reduces each pairwise computation on samples to features that were observed in both. The exact pairwise operations performed depend on the task under consideration. In this paper, we restrict our considerations to pairwise correlations, as they represent a particularly flexible class of metrics (e.g. rank correlation as opposed to correlation for continuous variables) which are by definition in the range $[-1,1]$. They provide a way to summarize relationships in an easily interpretable number and are commonly used by the biomedical community. As two representative examples, we consider Pearson correlation and Spearman’s rank correlation.

There are only few different classifiers capable of working with correlations in the commonly used vectorial representation among which we focus on the K-Nearest-Neighbor (KNN) classifier and the distribution-based classification (DBC) method.

The KNN algorithm [12, 13] is a classical, yet state-of-the-art approach which is capable of classifying observations by using those pairwise correlations (cf. [14, 15]). The KNN classifier assigns a class to a test instance by performing a majority vote among the K nearest neighbors. It thereby omits all additional information, such as (potentially meaningful) correlations to instances from other classes. Authors have introduced partitioning strategies, such as KD-tree and Ball-tree, that can accelerate the nearest-neighbor search [16, 17]. Since not all of them are applicable to correlations (eg. Ball-tree requires mathematical distance metrics), we restrict the considerations in this paper to the brute search for nearest neighbors.

Distribution-based classification (DBC) is a method introduced by Wei and Li [18] which compares similarity-score distributions within and between classes by means of the Kullback–Leibler (KL) distance. Among other metrics, they applied their method to pairwise correlations and found their approach to perform comparable or better than several other popular machine learning methods.

Although KNN and DBC both show excellent performance on some classification tasks, they offer very limited capabilities of being adapted to the data at hand. Our approach provides a modular concept with exchangeable baseline classifiers, each of which may be tuned specifically to the problem under consideration. For instance, a limited overlap of the expressed genes between two specific classes could render the correlation between samples from those classes essentially meaningless. Both KNN and DBC would still consider those correlations as equally important to other class combinations, whereas a RandomForest as baseline classifier would intrinsically assign lower feature importance to those meaningless values during the training process.

## Algorithm

In this section we describe the proposed method and compare our concept to the KNN and DBC classifiers. Furthermore, we introduce two modifications of our approach that allow to reduce the execution time and to neglect specific types of bias (e.g. batch effects).

The proposed method focuses on three key aspects:

- Tolerate close to arbitrarily many missing values without relying on imputation.
- Make use of (potentially discriminative) cross-correlations between classes (eg. instances from class A and B exhibit high mutual correlation, but while instances from A also have a high average correlation with instances from class C, those from class B don’t).
- Provide tuning options via modular components and parameters, allowing to even exchange the incorporated machine learning models.

**Fig. 1:**
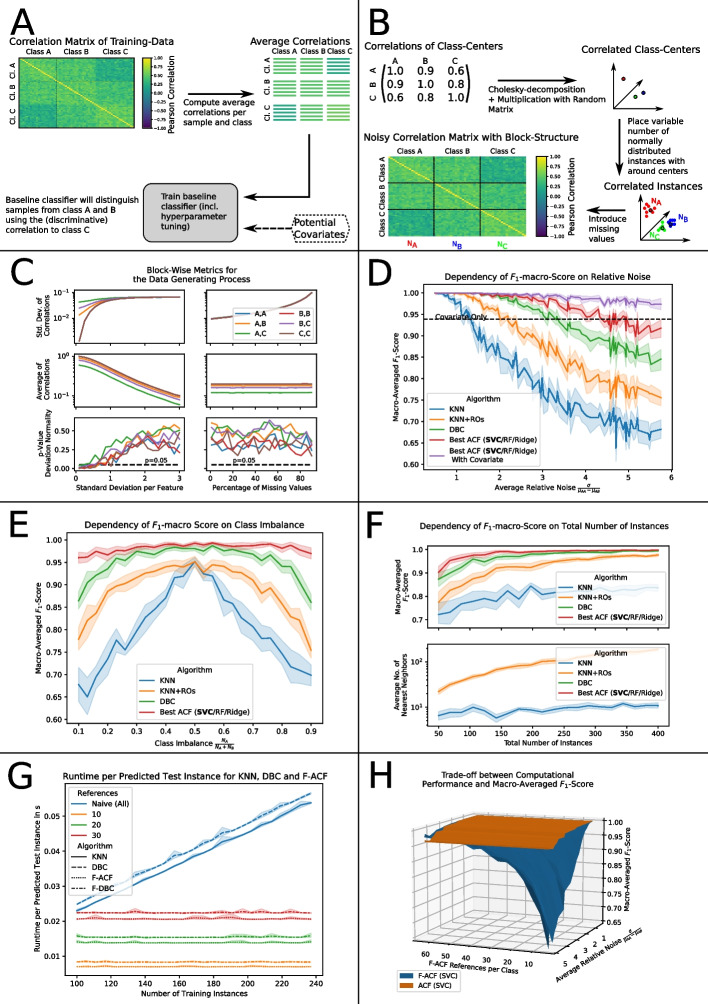
Concepts and results regarding the comparison of correlation-based classifiers on simulated datasets. **A** Concept sketch of ACF. **B** Concept sketch of the data-generating process for datasets with $N_{A}$, $N_{B}$, $N_{C}$ samples from each of the three classes A, B, C respectively. **C** Dependency of the properties of correlation matrices from the data-generating process on the percentage of missing values and the standard deviation per feature. Lines indicate the mean value per combination of classes A, B and C over 20 repetitions. **D** Dependency of the macro-averaged F1-score of ACF (red), DBC (green), KNN (blue) and KNN with random oversampling (KNN+ROs, orange) on the average relative noise. ACF may incorporate additional predictive covariates (violet). The predictiveness of the covariate alone is indicated in black. Solid lines and shaded areas indicate mean and standard deviations over 10 repetitions. For ACF, we tested multiple baseline classifiers (support-vector-classifier/RandomForest/ridge) and report the scores for the SVC which performed best in most cases. **E** Dependency of the macro-averaged F1-score (averaged over 10 repetitions) of the considered correlation-based classifiers on class imbalance. **F** Top: Dependency of the macro-averaged F1-score (averaged over 10 repetitions) of the considered correlation-based classifiers on the size of the training set. Bottom: Mean and standard deviation of the selected number of nearest neighbors for KNN and KNN+ROs. Results are averaged over 10 repetitions. **G** Mean and standard deviation over 10 repetitions of the runtime per predicted test instance for varying number of training instances. Linestyles indicate the respective algorithms (KNN, DBC, F-ACF and F-DBC) and colors represent the respective number of reference instances per class (10, 20, 30 and naïve (all)). For F-ACF, we chose a support-vector-classifier (kernel = “rbf”, C = 100) as baseline classifier. **H** Visualization of the trade-off between the number of reference instances for F-ACF and the F1-macro score achieved at a fixed noise level (averaged over 10 repetitions). Both ACF (orange) and F-ACF (blue) use a support-vector-machine as baseline classifier

In order to address all three aspects, we propose to fit tunable machine learning models to the empirical estimates for the average pairwise correlation between samples from each combination of classes (cf. Fig. 1A). We term this approach ACF (average correlations as features).

This method is particularly appropriate, if we assume the matrix $\textbf{C}_{Train}$ of all pairwise correlations between training observations to exhibit block structure upon ordering (cf. in “Discussion” section). This means, that the pairwise correlation $\textbf{C}_{Train}[s_{1}, s_{2}]$ between two samples $s_{1}$, $s_{2}$ is solely determined by their respective classes $C_{1}$, $C_{2}$, i.e.

1 $$\begin{aligned} \textbf{C}_{Train}[s_{1}, s_{2}] = \mu \left( C_{1}, C_{2}\right) + \epsilon , \end{aligned}$$

where $\mu (C_{1}, C_{2})$ denotes the expected correlation of samples from classes $C_{1}$ and $C_{2}$ and $\epsilon$ is a random variable that we assume to be normally distributed. In the most simplistic form, the proposed approach proceeds as follows (cf. Fig. 1A):

1. Compute the average correlations
  $$\begin{aligned} \mu (s_{i}, s_{k}), \quad y(s_{k}) = C, \quad k \ne i \end{aligned}$$
  of each training sample $s_{i}$ to all training observations $s_{k}$ per class *C* (self-correlations must not contribute, since they do not carry any information and would lead to biased estimates of the mean correlations).
2. Select a suitable classification model (e.g. RandomForest) and train it on the empirical estimates obtained in step 1. Additionally, other relevant covariates may be included, such as age or symptoms of a patient. The underlying classification model is also termed baseline classifier in the following. Common hyperparameter tuning approaches can be used to select and further adapt it to the problem.
3. Compute the average correlations of each test instance to all training instances per class.
4. Use the trained baseline classifier on the estimates obtained in step 3 (and all considered covariates) to predict the classes of the test instances.

In contrast to the KNN classifier, ACF intrinsically considers all cross-correlations between classes, without limiting itself to certain elements of $\textbf{C}_{Train}$. DBC also incorporates cross-correlations but relies on a fixed claiming-scheme and weighted Kullback–Leibler (KL) decision rules. For ACF, the baseline classifier may instead be chosen depending on the data and can be further adapted, e.g. increasing the depth of decision trees or applying regularization.

Both ACF and DBC require the computation of all pairwise correlations between training instances as well as between test instances and training instances. This raises important concerns regarding their computational performance, especially when compared to the KNN classifier that does neither require the computation of $\textbf{C}_{Train}$ nor a training step prior to prediction. Due to the computation of all pairwise correlations, the asymptotic time complexities of ACF are

2 $$\begin{aligned} \mathcal {O}_{Train}^{ACF}&= \mathcal {O}\left( n^{2}_{Train}\right) + \mathcal {O}_{Train}^{Baseline Classifier} \end{aligned}$$

3 $$\begin{aligned} \mathcal {O}_{Test}^{ACF}&= \mathcal {O}\left( n_{Train}n_{Test}\right) + \mathcal {O}_{Test}^{Baseline Classifier} \end{aligned}$$

for training and prediction respectively. $n_{Train}$ and $n_{Test}$ denote the number of instances in the training set and test set respectively whereas $\mathcal {O}_{Train/Test}^{Baseline Classifier}$ represents the time complexities of the baseline classifier for training and prediction.

Optimizing the time complexities of a particular baseline classifier is not within the scope of this paper, as the methodology is intended to work with arbitrary machine learning models. It is however possible to enhance the computational performance of ACF by estimating the average classwise correlations of a training instance using only a randomly selected subset of reference instances per class^1^ (cf. Additional file 2: Fig. S1, left panel). We term this faster variant of our algorithm F-ACF.

This approach is expected to yield coarser estimates of the average correlations, thereby coming with a trade-off in classification performance. Given a fixed number of reference samples and a baseline classifier with suitable time complexity, the prediction time of F-ACF is independent of the number of training instances. This differs from the prediction time complexity of the brute KNN classifier, which is at least linear in $n_{Train}$ due to the necessary $n_{Train}$ distance computations. This can conceptually not be reduced as it can for ACF.^2^ Therefore, our method brings additional flexibility in terms of time complexity and is particularly advantageous over the KNN classifier for large training sets.

Apart from computational performance, another relevant concern regards the existence of biased values in the correlation matrix. Such biased elements would also bias the obtained average correlations, which is why we propose to omit those biased correlations when performing the averaging (cf. Additional file 2: Fig. S1, right panel). As we assume block-structured correlation matrices (cf. Eq. 1), where the unbiased elements exhibit high redundancy, this does not affect classification performance as long as the number of biased elements remains relatively low. We refer to this modified algorithm as B-ACF.^3^ A particular weakness of this approach is that the exact location of the biased elements has to be known beforehand. As will be demonstrated in the “Results” section, this holds true for a simple model of batch effects in multiplexed proteomic measurements.

## Implementation

All studies have been conducted in Python. ACF, DBC and the KNN classifier are implemented as estimators compatible with current standards for machine learning modules. To ensure the correctness of our implementation, we validated our DBC-implementation against results from the original publication. Although other packages were used as well, the developed software and conducted analyses rely to large extent on the python-packages scikit-learn [19], optuna [20], numpy [21], scipy [22], pandas [23, 24], seaborn [25] and matplotlib [26]. All source code is publicly available at GitHub [27].

## Results

### Simulation studies

#### Consideration of classification performance

First, we analyzed the impact of noise present on the correlation matrix on classification performance. For this, we used the procedure described in the “Methods” section (cf. Fig. 1B, C) to generate simulated datasets of three classes with 70, 30 and 50 instances respectively. The first two classes (denoted as A and B in the following) were closely correlated but strongly differed in their correlation to the third class (denoted as C). Additionally, we generated an artificial covariate, which follows a Gaussian distribution with standard deviation 0.015 around the class centers at 0.15, 0.2 and 0.25 for A, B and C respectively.

We measure the reliability of class predictions (also referred to as classification performance in this paper) as the macro-averaged $F_{1}$-score, which is the harmonic mean of the average precision and average recall per class [28]. We report the dependency of the $F_{1}$-score on the average relative noise $\sigma _{rel} = \frac{\sigma }{\mu _{AA}-\mu _{AB}}$, averaged over 10 independent simulations. Here, $\sigma$ denotes the standard deviation of the noise on the correlation matrix and $\mu _{AA}$, $\mu _{AB}$ represent the average pairwise correlations between instances from A, A and A, B respectively. Although Spearman’s correlation works as well, we employ Pearson correlation for the simulation studies presented below, since we don’t expect strong outliers in the data and Pearson correlation will therefore yield unbiased estimates that capture more information than rank-based correlations.

As shown in Fig. 1D, the score of the KNN classifier decreased drastically with increasing relative noise. Using random oversampling improved the performance of the KNN classifier by allowing the hyperparameter optimization to select a higher number of nearest neighbors, which resulted in better averaging for the class prediction. For ACF, we tested three different baseline classifiers (support-vector-classifier/RandomForest/ridge) which yielded comparable $F_{1}$-scores. On average, the support-vector-classifier performed best. Both ACF and DBC maintain high $F_{1}$-macro scores even for much higher relative noise than the nearest-neighbor based approaches, which is most likely due to fact that they intrinsically consider cross-correlations. (While the average correlations $\mu _{AA}$, $\mu _{BB}$, $\mu _{AB}$, $\mu _{BA}$ might be indistinguishable at a relative noise of $> 1$, the discriminative cross-correlation with class C is only obscured at much higher noise, thereby allowing ACF and DBC to still yield reliable class predictions.) The $F_{1}$-score achieved by ACF exceeds the score of DBC, which we attribute to better adaption of the underlying support-vector-classifier via hyperparameter optimization for ACF instead of a fixed claiming-scheme as for DBC. Furthermore, ACF proved capable of further enhancing classification performance by considering additional covariates, as was demonstrated with the generated artificial covariate.

For all considered methods, the standard deviation of the macro-averaged $F_{1}$-score increased with the relative noise $\sigma _{rel}$. This is to be expected, since the computed correlations resemble coarser estimates of their true values, potentially moving samples closer to the decision boundaries of the corresponding classifier. At maximum $\sigma _{rel}$, the DBC classifier, KNN (with and without oversampling) and ACF without artificial covariate exhibited similar standard deviations of their $F_{1}$-scores (0.030, 0.040, 0.033, 0.035 respectively). Once the artificial covariate, which exhibited a constant standard deviation independent of $\sigma _{rel}$, was included, ACF achieved macro-averaged $F_{1}$-scores with considerably lower standard deviation than the other methods (0.019). At maximum relative noise, the average $F_{1}$-scores of each pair of methods were at least 1.50 standard deviations apart, indicating very robust results.

Further generated simulation data allows to discuss the effect of class imbalance on the performance of the respective classification approaches under consideration. We generated datasets of 150 instances with an average relative noise of $\sigma _{rel} = 2.9$. Class C had 50 observations, whereas the remaining samples were split between class A and class B with a varying ratio.

As depicted in Fig. 1E, all considered classifiers achieved their highest performance on balanced datasets. The $F_{1}$-macro score of the KNN classifier decreased drastically with increasing class imbalance. Using the KNN classifier with random oversampling to artificially balance the dataset yields equivalent performance in the balanced scenario, but mitigated the problem to a certain extent in the unbalanced scenarios, by allowing a larger number of nearest neighbors to be selected during hyperparameter optimization. This led to enhanced averaging of the class prediction, which countered the effect of noise to a certain degree. In the most strongly imbalanced scenarios, the average $F_{1}$-scores of KNN with and without random oversampling differed by at least 1.33 standard deviations.

Again, we observed only subtle differences between the $F_{1}$-scores of ACF with different baseline classifiers, but the support-vector-classifier (SVC) performed best. With this SVC as baseline classifier, ACF surpassed the performance of both KNN (with and without random oversampling) and DBC for all class ratios. The latter is of particular interest, since DBC is conceptually very robust to class imbalance, because all classes contribute equally regardless of their relative abundance. The robustness of ACF can be explained by the fact that many classical machine learning models, including the support-vector-classifier, offer balanced class weights as a possible hyperparameter. This allows the hyperparameter optimization procedure to ensure equal contribution of all classes during the training process of the baseline classifier, regardless of their respective number of instances.

While the difference between ACF and DBC is relatively small in the perfectly balanced scenario (at least 1.24 standard deviations), their difference is very pronounced in the strongly imbalanced scenarios (at least 3.02 standard deviations), indicating particularly robust results of the ACF approach.

Lastly, we expect the nearest-neighbors based approaches to be highly dependent on the total number of instances. To demonstrate this, we generated datasets of various sizes, each exhibiting relative class abundances of $\frac{7}{15}, \frac{3}{15}$ and $\frac{5}{15}$, as well as an average relative noise of $\sigma _{rel} = 2.6$.

Figure 1F reports the macro-averaged $F_{1}$-score in dependency of the total number of instances in the dataset. Among the different baseline classifiers, ACF performed best in combination with a support-vector-classifier. It is apparent that this combination of SVC and ACF outperformed the KNN classifier (with and without random oversampling) as well as DBC for small datasets. For medium-sized and large datasets, our approach exhibited a close-to-ideal $F_{1}$-macro score of approximately 1, whereas DBC and the KNN classifier with oversampling slowly converged towards this value. Without oversampling, the KNN classifier showed severely reduced scores, which can be attributed to the low number of considered nearest neighbors that was on average selected in the hyperparameter optimizations. With increasing size of the dataset, the macro-averaged $F_{1}$-scores of all considered methods exhibited a decreasing standard deviation. This is to be expected, since the higher number of training instances moves new samples away from the decision boundaries of the corresponding classifiers. In the considered scenarios, nearest-neighbor based approaches exhibited scores which were typically well separated from other methods by multiples of their respective standard deviations, while the difference between ACF and DBC was typically not as strongly pronounced.

#### Consideration of computational performance

In this section we compare the time complexities of the fast F-ACF algorithm, DBC and the (brute) KNN classifier. Furthermore, we compare the macro-averaged $F_{1}$-score of F-ACF and ACF.

To compare the asymptotic time complexities of the respective algorithms, we generated various datasets with 60 test instances and between 100 and 240 training instances. For DBC, we considered both the naïve DBC algorithm as described by Wei et al. [18], as well as a similar modification as in F-ACF, where the intra- and interclass distributions are approximated using only a fixed number of reference instances per class. We term this modified variant F-DBC. We selected a support-vector-classifier (C = 100, rbf kernel) as baseline classifier for F-ACF and tested F-ACF and F-DBC with 10, 20 and 30 reference instances per class.

Figure 1G reports the prediction time per test instance for each considered algorithm, averaged over 10 independent measurements. We expect the computation of pairwise correlations to dominate the runtime. This was confirmed experimentally by the observed linear scaling of KNN and the naïve DBC classifier, as well as the independent scaling for F-ACF and F-DBC. For all numbers of reference instances, F-ACF was faster than the corresponding F-DBC implementation. Furthermore, even without hyperparameter optimization for F-ACF, the average $F_{1}$-scores of F-ACF were in $65.3\%$ of the dataset configurations higher than the scores of the respective F-DBC algorithm (Additional file 2: Fig. S2, left panel). Employing hyperparameter optimization increases this ratio to $86.7\%$, but also increases the training time (Additional file 2: Fig. S2, right panel).

**Fig. 2:**
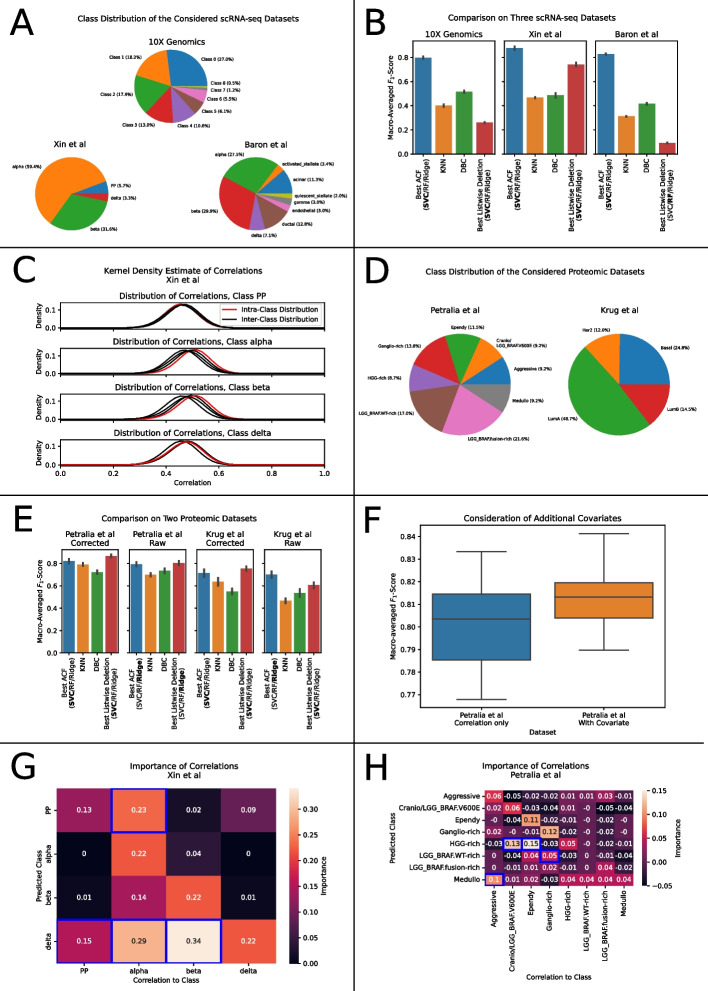
Characteristics of the considered datasets and approaches. **A** Class distributions of the considered scRNA-seq datasets. **B** Mean and standard deviations of the macro-averaged F1-scores of ACF (blue), KNN (orange), DBC (green) and conventional machine learning models (listwise deletion, red) on the three considered scRNA-seq datasets over 10 repetitions. For ACF and listwise deletion, we report the results for the baseline classifier maximizing the reported score. **C** Representative example of the approximated distributions of the correlations between samples from each pair of classes on the dataset by Xin et al. For the classes PP and delta (top and bottom), close similarities between inter- and intraclass distributions are observed. **D** Class distributions of the considered dataset from multiplexed proteomics. **E** Mean and standard deviations of the macro-averaged F1-scores of the considered classifiers on the considered proteomic dataset over 10 repetition. Scores are reported for raw and for batch-corrected (IRS) data. **F** Representative example of the improved prediction of proteomic subtypes on the dataset by Petralia et al. when considering histopathologic diagnoses. Scores refer to the baseline classifier maximizing the classification performance. **G**, **H** Importance of average class-wise correlations for the prediction of each class for the datasets by Xin et al. (**G**) and Petralia et al. (**H**). Variable importance was measured by the decrease in class-wise F1-score of an unoptimized support-vector-classifier (C = 100, kernel = rbf, balanced class weights) when removing the considered correlation

The observed independent scaling as well as the high $F_{1}$-scores compared to F-DBC demonstrate that F-ACF might be particularly suited for classification tasks with large training sets.

Reducing the number *n* of reference instances per class decreases prediction time, but will generally yield coarser estimates for the average correlations, thereby leading to lower classification performance of F-ACF. Figure 1H illustrates the trade-off between the number of reference instances per class and the achieved $F_{1}$-macro score at various noise levels. Furthermore, we also report the $F_{1}$-macro score of the ACF algorithm at the same relative noise. For comparability, both algorithms used a support-vector-classifier as baseline classifier.

At low relative noise, a small number of considered instances per class was sufficient to yield reliable estimates of the average correlations (and therefore a high $F_{1}$-macro score of F-ACF). Higher relative noise however required a larger number of references to yield comparable classification performance. Unsurprisingly, the highest macro-averaged $F_{1}$-score was obtained using all accessible instances per class (ACF). In an application, the number of reference instances required by F-ACF to achieve good classification performance would be determined automatically using a hyperparameter optimization library, such as optuna [20].

### Comparison with KNN, DBC and conventional machine learning methods on biologic datasets

The results from the previous section were based on simulated datasets that were engineered to exhibit discriminative cross-correlations. However, the benefit of applying our model has still to be demonstrated on real-world datasets. In this section, we apply our approach^4^ to datasets from scRNA-seq and proteomics and validate it against KNN, DBC and established, conventional machine learning models. For the latter, we consider the models that were used as baseline classifiers for ACF and directly apply them to the gene expression data, handling the missing values using listwise deletion.

We considered datasets from three scRNA-seq experiments by Baron et al. [29], Xin et al. [30] and 10XGenomics [31]. The respective class distributions of the datasets are schematically summarized in Fig. 2A. Further information on the datasets can be found in Additional file 2.

Figure 2B reports the respective macro-averaged $F_{1}$-scores obtained on the three scRNA-seq datasets. Since listwise deletion removed all genes on the dataset by Baron et al, the reported scores of the conventional machine learning models on that dataset were determined by random class assignment.

The proposed approach, ACF, strongly outperformed the other methods with significant differences on all three datasets, regardless of the selected baseline classifier (cf. Additional file 1 and Additional file 2: Table S6). The differences between different baseline classifiers for ACF were not always significant, although a support-vector-classifier generally appeared to be a good choice. The three correlation-based approaches, ACF, KNN and DBC, outperformed the combination of conventional machine learning methods with listwise deletion on two out of three datasets. This demonstrates that our initial motivation of avoiding data loss was highly reasonable in the context of imputation-free classification. Although DBC generally had better $F_{1}$-scores than KNN, the difference between these two methods was rather small. We attribute this to the indistinguishability of inter- and intra-class distributions for many classes on the datasets (cf. Fig. 2C). This reduces the probability for true positives in the claiming-scheme of DBC and makes false positives more likely at the same time. This explanation is supported by the fact that we also observe reduced precision and recall of DBC for these classes (cf. Additional file 2: Table S8 for an example).

The proteomic datasets differ from the scRNA-seq datasets in multiple ways: Methods such as isobaric labelling of peptides and other technologies can increase the number of identified peptides so that the missing value problem is not as prevalent as in scRNA-seq data. Furthermore, combining several multiplexed experiments introduces a bias (the so called batch effect) among instances from different experiments. The existence of such biases poses a major challenge for biologic analysis as well as classification. Common techniques to correct batch effects include the usage of internal reference samples (IRS) between experiments [32] and the application of batch effect correcting algorithms such as ComBat [33]. In this study, we employ internal references for batch correction.

We consider two proteomic datasets by Petralia et al. [34] and Krug et al. [35]. Their respective class distributions are visualized in Fig. 2D. Further details on the datasets are provided in Additional file 2.

Figure 2E reports the respective macro-averaged $F_{1}$-scores achieved by the individual classification models. For the batch-effect corrected data (Fig. 2E, 1st and 3rd column), the combination of conventional machine learning models with listwise deletion performed best, closely followed by ACF which yields significantly higher classification performance than the other correlation-based approaches (cf. Additional file 1 and Additional file 2: Table S7). For the uncorrected data (Fig. 2E, 2nd and 4th column), we employ B-ACF and a similarly modified version of DBC. For this, we modeled the batch effect to bias only the correlations of samples from the same batch (cf. Additional file 2: Fig. S3), which is a simplistic approximation, but proves to be powerful by enhancing classification performance. This flexibility is however not offered for the KNN classifier that only works on the entire (unmasked) correlation matrix. On this unadjusted data, B-ACF outperformed both KNN and the modified DBC with significant differences (cf. Additional file 2: Table S1). This supports our simplistic model of the batch effect and demonstrates the flexibility of the classification approach presented here.

The modularity of ACF even allows to integrate deep-learning based methods, such as a multi-layer perceptron (MLP) as baseline classifier. As proof-of-concept, we conducted experiments on one exemplary scRNA-seq dataset and proteomic dataset each, where we tested a MLP as baseline classifier and compared the results for the deep neural network with the conventional baseline classifiers discussed before (cf. Additional file 2: Fig. S4). The results indicate that using a deep neural network as baseline classifier may offer a slight improvement over the previously discussed conventional machine learning methods.

While KNN and DBC intrinsically rely on the pairwise correlations exclusively, ACF offers the flexibility to incorporate further covariates into the classification process. This advantage of ACF is demonstrated by the observation of improved $F_{1}$-scores, when considering histopathologic diagnoses as covariate for the data by Petralia et al (Fig. 2G).

To estimate the variable importance of each average correlation for the prediction of individual classes, we selected a support-vector-classifier with typical hyperparameters (C = 100, balanced class weights, rbf-kernel) as baseline classifier for ACF and employed repeated stratified cross-validation. We measured the decrease of the $F_{1}$-score for each class, when individual average correlations were not passed to the baseline classifier. We observed discriminative cross-correlations (variable importance $> 0$ for the average correlations to samples from other classes) on all considered datasets from each omic type (cf. Fig. 2G, H). This highlights the importance of considering *all* correlations when using absolute correlation values (as in ACF and DBC) instead of relative values, such as for KNN.

## Discussion

The aim of this study is to explore the use of pairwise correlations for classification based on molecular data. This is motivated by the widespread use of correlations in the biomedical community as well as the fact, that they allow to summarize relationships in an easily interpretable number.

Previous work on correlation-based classification is scarce, but researchers have used the K-Nearest-Neighbor (KNN) classifier and DBC (distribution based classification) [14, 15, 18]. With ACF, we present a novel method for correlation-based classification, which can be flexibly adapted to a large number of settings. By using pairwise metrics, it works in an imputation-free fashion, whilst minimizing data loss.^5^ While the KNN classifier only considers the *k* highest correlations, both ACF and DBC intrinsically consider cross-correlations. DBC however relies on a fixed claiming-scheme, whereas ACF offers the flexibility of choosing and adapting tunable classification models to the data under consideration.

This makes ACF particularly suitable for the application to datasets with large portions of missing values, such as from dataset integration [3]. Candidate problems include, but are not limited to, multi-omic datasets as well as datasets assembled from multiple laboratories, leading to various kinds of missing values.

The computation of pairwise correlations relies on pairwise deletion, which is rarely used compared to listwise deletion and imputation. Based on our results, we see great potential in both the application and future research on approaches based on pairwise deletion (see “Conclusion and Outlook”). ACF makes use of average correlations, whereas DBC employs the Kullback–Leibler distance between distributions, thereby capturing further potential information, such as skewness. However, we observed significantly reduced scores when combining ACF with Kullback-Leibler distances. We attribute this to the unfavorable divergence of Kullback–Leibler distance, which makes strong outliers more likely. Therefore, we focused on average correlations only, although our implementation allows the user to select other metrics such as median correlations.

The simulation studies we conducted show that the proposed method yielded much higher macro-average $F_{1}$-scores than the KNN classifier for noisy, small or imbalanced datasets and also performed comparable or better than the DBC method. We tested different baseline classifiers for ACF which all performed comparably well on the simulated datasets, but in the majority of cases considered in this study, a support-vector-classifier yielded slightly more favorable scores than the other methods. Additional proof-of-concept experiments indicate that using deep-learning methods (e.g. MLPs) as baseline classifiers may yield slight improvements over conventional machine learning methods. Whilst this manuscript focuses on presenting the method and applying three conventional machine learning methods as baseline classifier, an optimal baseline classifier (e.g. MLP) may in practice easily be selected as part of the hyperparameter optimization procedure.

The process used for generating the simulated datasets (cf. “Methods” section) was developed to allow precise control over the block-structure and the noise of the correlation matrix and is based on the introduction of missing values to instances that are normally distributed around correlated class centers. We argue, that this procedure is highly reasonable in a biological context: Firstly, the high dimensionality as well as the high number of missing values is common in many datasets, e.g. in scRNA-seq experiments, cf. [37]. Secondly, assuming instances to be normally distributed around a class-specific center is reasonable, as many relevant sources of variation, e.g. measurement error, can be approximated to be normal. Lastly, correlation matrices from biologic datasets commonly exhibit block-structure (cf. [38] for an example), in which some of the cross-correlations may allow class discrimination. Our findings suggest that this phenomenon occurs commonly in real-world datasets, i.e. from scRNA-seq, which in turn shows that it is reasonable for the data-generating process to generate datasets with such discriminative cross-correlations.

We also demonstrated that ACF offers the flexibility to be modified in such a way that the time complexity for prediction is independent of the number of training instances (F-ACF), whereas it scales approximately linearly for the KNN classifier. The same modification is possible for DBC, but yielded both less efficient as well as less accurate predictions than F-ACF.

We showed on data from three scRNA-seq experiments, that our approach significantly outperformed both KNN and DBC as well as listwise deletion combined with several conventional classifiers (RandomForest, SVM, Ridge). On datasets from proteomics, ACF yielded better $F_{1}$-macro scores than the other correlation-based classifiers, especially when incorporating a simplistic model for batch effects.

In summary, this work explores and compares different approaches to correlation-based classification. The proposed approach, ACF, offers peculiar advantages, such as tolerance to missing values, the consideration of cross-correlations as well as potential covariates, and the capability of being adapted to the considerd dataset by means of hyperparameter optimization. Our results demonstrate superior classification performance of ACF over established correlation-based techniques in extensive simulation studies, as well as on biologic datasets from scRNA-seq and proteomics.

The assumption of block-structured correlation matrices as well as the dependence on approximate average correlations constitute two important limitations to ACF: While the former is required to establish average correlations as meaningful metrics for classification, the latter implies that the correlation between samples from two classes may not vary too strongly relative to the number of samples used for averaging, in order to obtain meaningful averages. We found the considered real-world datasets to satisfy both requirements.

It is important to note that this paper focused entirely on the comparison of ACF, DBC and KNN in an imputation-free setting. We explicitly excluded imputation methods from the considerations here, since we were concerned about their generally weak generalization across different omics types and datasets as well as their applicability in presence of different types of missing values [4–6].

## Conclusion and outlook

We presented our novel correlation-based classification approach ACF. The particular advantage of ACF lies in the combination of tolerance to missing values, consideration of cross-correlations and the capability of providing tuning options via modular components and parameters. In simulation studies, we found our approach to work particularly well when considering small, imbalanced or noisy datasets, which are challenging for most algorithms. We observed statistically significant improvements over KNN and DBC on experimental data from two representative omics-technologies, namely scRNA-seq and proteomics. Furthermore, we demonstrated that ACF offers high flexibility with respect to time complexity and modeling of certain biases (e.g. batch effects), thereby enabling problem-specific adaptions to various applications.

Directions of further research include the evaluation of ACF on multi-omics datasets as well as the comparison of ACF with deep learning models. For the latter, a particularly interesting approach might be to adapt the recently published DeepOmicNet architecture to classification tasks, which would allow for highly efficient training due to the usage of grouped bottleneck structures and skip connections [39]. Other architectures of interest include, but are not limited to, the multi-layer perceptron and deep-belief networks [40].

## Methods

### The ACF method

The proposed method (Average Correlations as Features, ACF) aims to provide tolerance to missing values, consideration of cross-correlations, as well as the capability of being flexibly adapted to the data at hand. This is achieved by fitting tunable machine learning models to empirical estimates for the average pairwise correlations between samples from each combination of classes.

The procedure starts by computing the average correlations of each training sample to all other training observations per class. (Depending on the considered problem, other empirical metrics, such as the median, may be used as well. In this study however, we focus on average correlations exclusively.) In the next step, a machine learning model (referred to as baseline-classifier) is trained to predict class labels based on the previously obtained averages and potential other covariates, such as the age of a patient (cf. Fig. 1, panel A). For the classification of a test sample, the trained baseline classifier is provided with all potential covariates as well as with the average correlations between the test sample and the previously considered training samples per class.

The computational performance of ACF may be enhanced by estimating the average correlation based on less training samples. This approach is referred to as F-ACF throughout this manuscript. This method reduces the required number of computations, but may result in coarser estimates of the average correlations which can reduce classification performance. If specific correlations between samples are known to be biased (e.g. due to batch-effects), these correlations may be masked out during the computation of the empirical averages. This modified approach is referred to as B-ACF throughout the manuscript.

For a motivation of the method, as well as further details, we refer the reader to the “Algorithm” section.

### Data-generating process

We aimed to generate data with block-structured correlation matrices and discriminative cross-correlations with sufficient control over

- the overall block-structure of the correlation matrix,
- the standard deviation of the (Gaussian) noise $\sigma$ on the correlation matrix
- as well as the number of classes, the number of instances per class and the size of the dataset.

Meeting these requirements, we developed a data-generating process (DGP) that is based on the introduction of missing values to high-dimensional data points which are normally distributed around points that were chosen to exhibit a specified correlation matrix (see Fig. 1B). This DGP, which was used to generate datasets for all simulation studies in this paper, proceeds as follows:

We choose the dataset under consideration to exhibit 10,000 features and to consist of instances from three classes. This corresponds to the minimum number of classes that can exhibit a discriminative cross-correlation. The centers of those classes are expected to be correlated with a correlation matrix of

4 $$\begin{aligned} \textbf{C}_{Centers} = \begin{pmatrix}1.0&{}\quad 0.9&{}\quad 0.6\\ 0.9&{}\quad 1.0&{}\quad 0.8\\ 0.6&{}\quad 0.8&{}\quad 1.0\end{pmatrix} \end{aligned}$$

Throughout this paper we focus on this specific matrix, since it provides a minimal example of discriminative cross-correlations. (The centers for the first two classes are closely correlated but strongly differ in their correlation to the third class.) Considering variations of the individual correlations in Eq. (4) might offer the opportunity for further characterization in future works, but is clearly not in the scope of this paper, since they can potentially affect the noise distribution on the final correlation matrix and would therefore need to be chosen very carefully (see elaboration below).

By applying a Cholesky-decomposition [41] to $\textbf{C}_{Centers}$ and multiplying the resulting matrix with a suitably shaped random matrix drawn from a multivariate standard distribution, we obtain centers with approximately the specified correlation matrix. The final observations are then drawn from multivariate Gaussian distributions around each of the previously created centers, where the standard deviation of all Gaussian distributions is controlled via the parameter $\sigma _{Feature}$. The number of samples that are drawn per distribution determines the number of instances per class and correspondingly also the size of the dataset. Finally, we introduce a fixed percentage of completely randomly missing values to each observation.

The correlation matrix of the resulting dataset follows the block-structure specified by $\textbf{C}_{Centers}$. We observed that the parameter $\sigma _{Feature}$ introduces a scaling factor to the correlation matrix (cf. Fig. 1C, left center), which is to be expected, since $\sigma _{Feature}$ determines the standard deviation per feature, thereby reducing the correlation of each pair of instances. Furthermore, the percentage of missing values determines the standard deviation of the noise (denoted $\sigma$ in the following) on the correlation matrix (cf. Fig. 1C, top right). This is understandable, since missing values cause the pairwise correlation of each pair of samples to be computed using only a subset of features, thereby introducing variance to the individual correlations.^6^ Since each pairwise correlation must be in the interval $[-1,1]$, we were initially concerned about our assumption that the noise on the correlation matrix is Gaussian (which is an unbounded distribution). Using the test from D’Agostino and Pearson [42] we found however, that suitably low average correlations allowed for very high standard deviations $\sigma$ without significant deviation from normality (cf. Fig. 1C, bottom left and bottom right). (Such low values can either be achieved by variation of $\sigma _{Feature}$ or $\textbf{C}_{Centers}$.) Furthermore, we found that the value of $\sigma$ of the individual blocks of the correlation matrix were all equal and increased non-linearly with the percentage of missing values. Finally, we could show empirically, that the parameter $\sigma _{Feature}$ did not impose any changes on the block-wise standard deviations as long as the values in the correlation matrix were low enough so that the range of the correlation values $[-1,1]$ did not conflict with normality of the noise (cf. Fig. 1C, top left). If $\sigma _{Feature}$ was very low, the elements of the correlation matrix became close to the boundaries for correlations, hence the noise could not be symmetric anymore. Throughout this paper, we chose $\sigma _{Feature}=2.0$ which allows a suitable range of missing values rates without violating the assumption of normality.

### Machine learning methodology

We measure the reliability of class predictions (also referred to as classification performance in this paper) as the macro-averaged $F_{1}$-score, which is the harmonic mean of the average precision and average recall per class [28]. Since all classes contribute equally to the averaging, it is particularly insensitive to the class imbalance that often occurs in biologic datasets, including the ones considered in this paper.

Especially the considered proteomic datasets are small with only as few as $<20$ samples per class, rendering it infeasible to hold out a sufficiently large, representative test set for accurate evaluation of the considered methods. We therefore employ stratified, 10-fold cross-validation to measure the macro-averaged $F_{1}$-score on the considered datasets.

Using this approach, the dataset is first randomly split into *k* disjoint sets of samples (throughout the manuscript, it is $k=10$). Since the datasets under consideration exhibit considerable class-imbalance, each of the sets is constructed to approximately preserve class frequencies, which helps to reduce experimental variance [43]. In a round-robin like fashion, one set is then held out as test set for evaluation, while the remaining $k-1$ sets are used for the training procedure of the corresponding classifier. In particular, the test set is not used during the training or validation procedure and the evaluated classifiers are fully independent.

While DBC resembles a parameter-free algorithm and may be trained directly on the k − 1 sets, both KNN and ACF (as well as the underlying baseline classifiers themselves) allow for hyperparameter optimization during the training procedure. For the hyperparameter optimization, we sample candidate parameter values using a tree-structured Parzen estimator [44, 45]. For each parameter combination, 10 validation sets of 10% size are independently and randomly drawn from the union of the $k-1$ splits. For each of the validation sets, a classifier with the considered parameter combination is trained on the remaining data. The parameter combination is then assigned the mean macro-averaged F1-score of the classifiers on the respective validation sets. After 60 iterations of hyperparameter optimization, the best parameter combination is selected and used to train a new classifier on the full $k-1$ splits. Finally, this new classifier is evaluated on the respective held-out test set. This procedure is repeated for each of the *k* sets.

To account for experimental variations, e.g. during hyperparameter optimization, the entire cross-validation procedure is repeated 10 times, resulting in a mean macro-averaged F1 test-score that we report per dataset.

For ACF and its variants, we optimize various hyperparameters for different baseline classifiers: For a support-vector-classifier, we optimize the kernel (linear/rbf), the regularization parameter $C\in [5 \times 10^{-3}, 5 \times 10^2]$, the kernel coefficient $\gamma$ for rbf-kernels (scale/auto) and the class weights (balanced/None). For a RandomForest, we choose the number of decision-trees to be between 80 and 300, their depth between 2 and 40, the number of features within the entire possible interval and the class weight to be either balanced or None. The Ridge-Classifier is optimized using the regularization strength $\alpha \in [10^{-3}, 10^{4}]$ and the class weight (balanced/None). In our proof-of-concept experiments employing a multi-layer perceptron (MLP) as baseline classifier, we set the number of epochs to 400 and optimize the initial learning rate $\lambda \in [10^{-4},10^{-2}]$, the learning rate scheduling (constant/adaptive), the activation function (tanh/ReLU/logistic), the $L_{2}$ regularization strength $\alpha \in [10^{-4},10^{-1}]$ and the number of hidden layers between 1 and 40.

When reporting the respective $F_{1}$-scores of the conventional machine learning methods (SVC/RandomForest/Ridge, without ACF), we optimize the same set of hyperparameters as above. All other parameters, which are not optimized, are set to their default values in the scikit-learn library [19].

For the KNN classifier, we include the number $K \in [1, n_{Train}]$ of considered nearest neighbors in the optimization process, as well as their weights (uniform/distance-based). DBC is parameter-free and does not require any hyperparameter optimization.

We use a corrected right-tailed paired t-test for pairwise comparison of the classification performance of all considered models. A Bonferroni-correction is employed to correct for multiple testing (cf. [46]).

## Supplementary Information

**Additional file 1.** Table with p-values for pairwise comparisons between the tested classification approaches on the 5 biologic datasets.

**Additional file 2.** Supplementary Information.

## Data Availability

The code for this study as well as the implementation for the ACF-algorithm is publicly available on the web at [27]. There, we also provide documentation, examples and all raw data generated by the classifiers on the individual datasets. The biologic datasets considered in this study have been made publicly available by the respective authors of the original studies (see Additional file 2 for accession numbers and references to the online archives).

## Footnotes

1 This does not reduce the size of the training set, but only the number of correlations used for estimating the average correlations per training instance. Reducing the number of computed pairwise correlations for the KNN classifier on the other hand would intrinsically require reducing the total size of the training set.

2 A similar modification is possible for DBC, this has however not been explored in the original study [18].

3 A similar modification is possible for DBC, this has however not been explored in the original study [18].

4 In contrast to the simulation studies presented above, biologic datasets may easily contain strong outliers which could bias the estimates for the pairwise Pearson correlations. Therefore, we employ Spearman’s rank correlation on the real-world datasets because rank-based correlations are more robust to outliers.

5 Unlike many other statistical methods, neither ACF, KNN nor DBC require the correlation matrix to be positive definite, which is a general concern associated with pairwise deletion (cf. [7, 36]).

6 Introducing missing values with a MNAR mechanism would introduce a bias instead of noise, since the subset of features used for the computation of a pairwise correlation would be determined by the classes of the considered samples.
