# Supplementary material for: Robust classification using average correlations as features (ACF)
Source: BMC Bioinformatics. 2023 Mar 20;24:101. doi: 10.1186/s12859-023-05224-0 (PMC10026437; doi:10.1186/s12859-023-05224-0)
Supplement: Supplementary file 2 — Additional file 2. Supplementary Information. [file 12859_2023_5224_MOESM2_ESM.pdf]

Supplementary Information

1 Concept of ACF, F-ACF and B-ACF

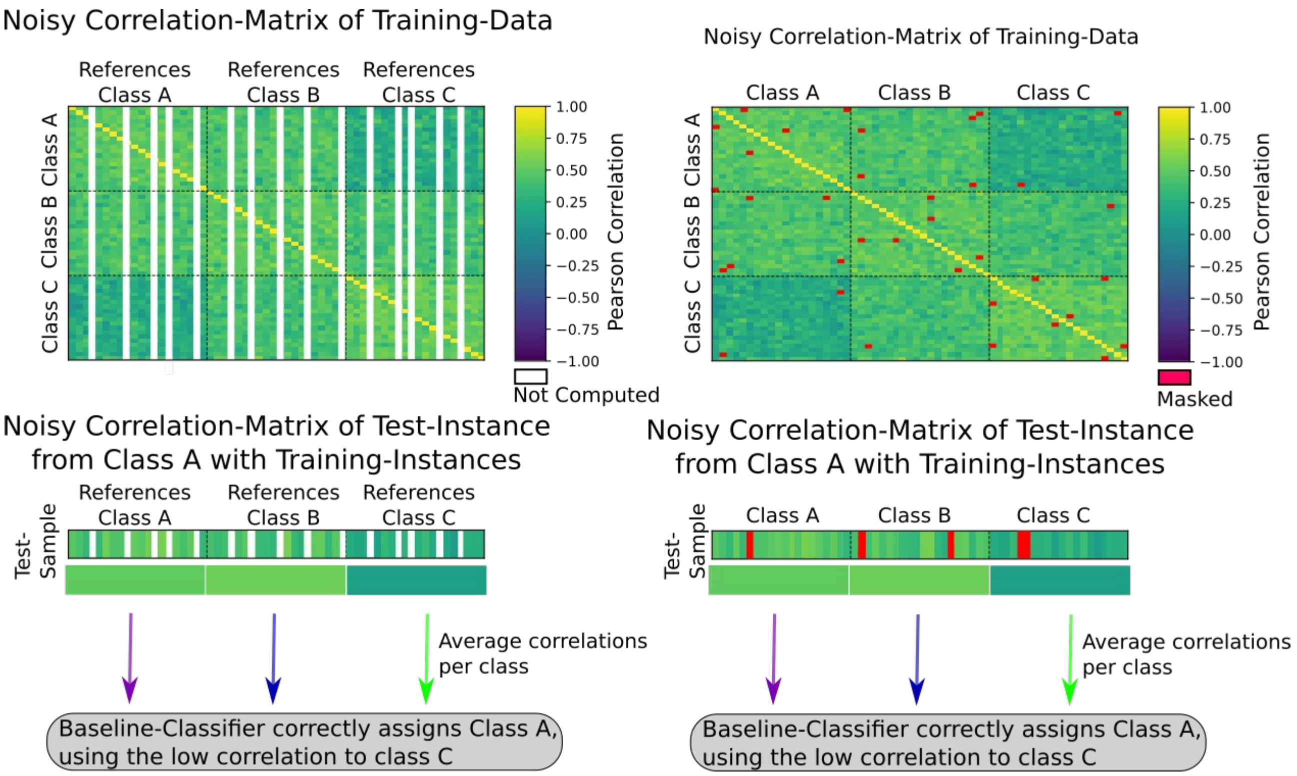

Figure 1: Concept of F-ACF (left) and B-ACF (right). F-ACF uses only a subset of reference instances to estimate the average correlations per class, thereby reducing the number of computed correlations. B-ACF masks biased correlations of both training instances and test instances and therefore uses unbiased estimates for the average correlations. Note that for clarity of representation, the respective training steps and preprocessing steps are not included in this figure.

2 Comparison of F-ACF and F-DBC

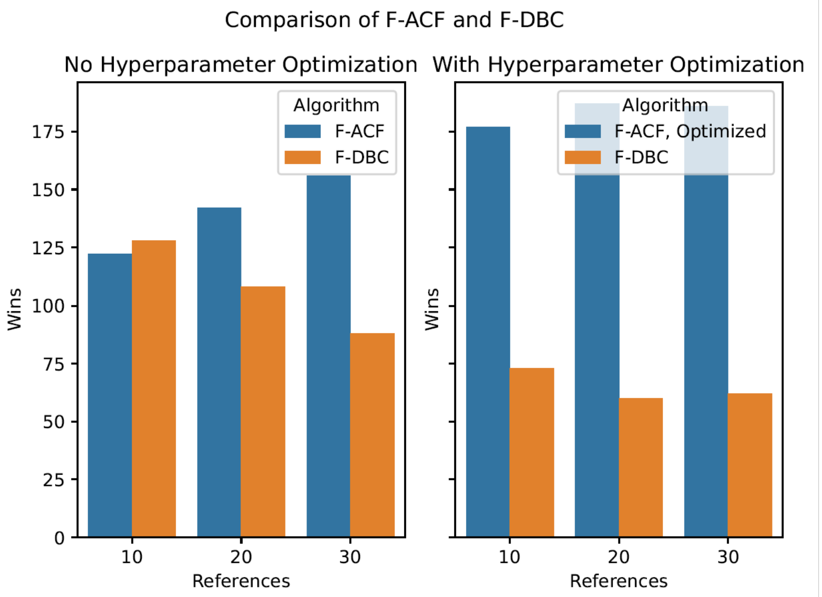

Figure 2: Comparison of F-ACF and F-DBC without hyperparameter optimization (left) and with hyperparameter optimization (right). On 250 generated datasets, the win rate (higher F_1-scores) of F-ACF over F-ACF increases from 65.33% to 86.67%when employing hyperparameter optimization.

3 Considered Datasets

All datasets considered in this study have been made publicly available by the authors of the corresponding studies. This section describes general properties associated with the data.

3.1 ScRNA-seq Datasets

3.1.1 10X Genomics

This dataset consists of 2700 peripheral blood mononuclear cells (PBMCs) from a healthy donor and has been published by 10X Genomics on 26th of May, 2016 under the Creative Commons Attribution license. We followed the Guided Clustering Tutorial of Seurat (https://satijalab.org/seurat/articles/pbmc3k_tutorial.html, last visited on 23rd of February, 2022) to assign classes to the cells.

We used Seurat V4.0.2 to eliminate cells with more than 2500 and less than 200 unique feature counts, as well as cells with a mitochondrial contamination of ≥5%. This left us with 2638 cells and a total of 32738 unique features.

We selected the 2000 most variable features, before scaling the data and performing a PCA. The first 10 principal components were selected for further analysis.

Using a KNN-graph based clustering approach, Seurat assigns each of the cells to one of 9 clusters with the following class distribution

Table 1: Number of cells per class in the scRNA-seq dataset by 10X Genomics.

Class	Number of Cells
0	711
1	480
2	472
3	344
4	279
5	162
6	144
7	32
8	14

3.1.2 Baron et al.

This dataset has been published by Baron et al. in 2016 in their publication "*A Single-Cell Transcriptomic Map of the Human and Mouse Pancreas Reveals Inter- and Intra-cell Population Structure*" and is accessible via GEO accession number GSE84133. We only consider the cells from the 4 human donors.

We retrieved the dataset using the R-library scRNAseq by Risso and Cole (2021), V2.4.0. We set zeros to NaN and export the UMI counts. The resulting dataset consisted of 8569 cells from 14 classes and with 20125 features.

In order to enable stratified subsampling of the dataset, we excluded classes that don’t make up at least 1% of the total number of cells. Then, we randomly selected a stratified subset of 40% from the dataset.

The final dataset consisted of 3380 cells with all 20125 genes and the class distribution summarized in Table 2.

Table 2: Class distribution for the dataset by Baron et al.

**Class**	**Number of Cells**
acinar	383
activated_stellate	114
alpha	930
beta	1010
delta	240
ductal	431
endothelial	101
gamma	102
quiescent_stellate	69

3.1.3 Xin et al.

This dataset of pancreatic cells from 18 donors has been published by Xin et al. in 2016 in their publication “*RNA Sequencing of Single Human Islet Cells Reveals Type 2 Diabetes Genes*” and is accessible via GEO accession number GSE81608.

We retrieved the dataset using the R-library scRNAseq by Risso and Cole (2021), V2.4.0. We set zeros to NaN, exclude all contaminated cells and export the RPKM values. The resulting dataset consisted of 1492 cells and 39851 genes.

The resulting class distribution is summarized in Table 3.

Table 3: Class distribution for the dataset by Xin et al.

**Class**	**Number of Cells**
PP	85
alpha	886
beta	472
delta	49

3.2 Proteomic Datasets

3.2.1 Petralia et al.

This dataset has been published by Petralia et al. in their publication “*Integrated Proteogenomic Characterization across Major Histological Types of Pediatric Brain Cancer*” from 2020. It contains 218 samples of pediatric brain tumors that were analyzed using MS3 and liquid chromatography. The raw proteomics data and processed proteogenomics data is publicly available on the web at https://cptac-data-portal.georgetown.edu/cptacPublic/. Raw data was processed with MaxQuant and 9155 proteins were quantified in total.

The authors of the original publication assign 8 proteomic subtypes with the class distribution summarized in Table 4.

Table 4: Class distribution for the dataset by Petralia et al.

**Class**	**Number of Samples**
Aggressive	20
Cranio/LGG_BRAF.V600E	20
Ependy	25
Ganglio-Rich	30
HGG-rich	19
LGG_BRAF.WT-rich	37
LGG_BRAF.fusion-rich	47
Medullo	20

3.2.2 Krug et al.

This dataset has been published by Krug et al. in their publication "*Proteogenomic Landscape of Breast Cancer Tumorigenesis and Targeted Therapy*" from 2020. 125 primary, treatment-naive breast cancers were analyzed using LC-MS/MS. Both raw and characterized data is publicly available at https://cptac-data-portal.georgetown.edu/study-summary/S060. We obtained the processed dataset directly from the authors of the study. 13769 proteins were quantified in total.

The targeted class in our study is the PAM50 type. We excluded tumors of the "Normal"-type, since only 6 samples of this type were measured. The resulting class distribution is summarized in Table 5.

Table 5: Class distribution for the dataset by Krug et al.

**Class**	**Number of Samples**
Basal	29
Her2	14
LumA	57
LumB	17

4 Considered Model for Batch Effects

We model the batch effect associated with the two proteomic datasets as depicted in Figure 2. Our model assumes, that the only correlations affected by the batch effect are the correlation between samples from the *same* batch. All other correlations remain unaffected.

Those correlations can be masked using B-ACF.

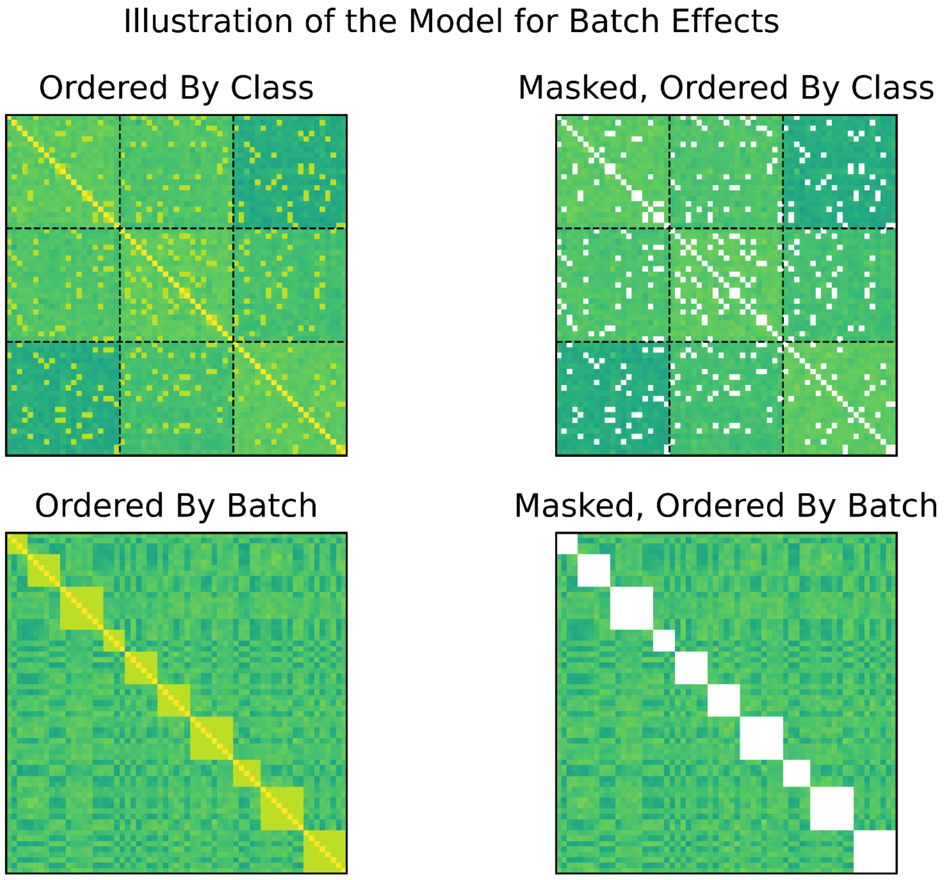

Figure 3: Illustration of the batch effect model. The batch effect biases only the correlations between samples from the same batch. This can be masked by B-ACF.

5 Results of the Individual Classifiers on the Biologic Datasets

For transparency reasons we report the individual F_1 scores of the respective classifiers on the datasets from scRNA-seq. (cf. Table 6) and multiplexed proteomics (cf. Table 7).

ACF+RF, ACF+Ridge and ACF+SVC indicate our ACF method with a RandomForest, Support-Vector-Classifier and Ridge-Classifier as baseline classifier respectively.

The scores reported for RF, Ridge and SVC resemble the performance of the indicated classifier based on listwise deletion.

Furthermore, we provide the classification report for DBC on the dataset by Xin et al (cf. Table 8). The results are based on 5-fold, stratified cross validation.

Table 6: Mean and standard deviation of the F_1 scores of the individual classifiers on the scRNA-seq datasets.

**Algorithm**	**3kPBMC**	**Baron**	**Xin**
ACF+RF	0.769+-0.054	0.801+-0.034	0.778+-0.091
ACF+Ridge	0.725+-0.049	0.797+-0.021	0.841+-0.085
ACF+SVC	0.801+-0.048	0.821+-0.031	0.803+-0.083
DBC	0.515+-0.05	0.416+-0.036	0.487+-0.096
KNN	0.406+-0.051	0.315+-0.01	0.465+-0.018
RF	0.251+-0.016	0.091+-0.015	0.623+-0.072
Ridge	0.151+-0.014	0.095+-0.016	0.739+-0.077
SVC	0.259+-0.018	0.091+-0.016	0.738+-0.084

Table 7: Mean and standard deviation of the F_1 scores of the individual classifiers on the scRNA-seq datasets.

**Algorithm**	**Krug (IRS)**	**Krug (raw)**	**Petralia (IRS)**	**Petralia (raw)**
ACF+RF	0.723+-0.144	0.667+-0.146	0.832+-0.076	0.781+-0.111
ACF+Ridge	0.682+-0.155	0.702+-0.144	0.809+-0.091	0.795+-0.091
ACF+SVC	0.707+-0.131	0.663+-0.148	0.83+-0.089	0.804+-0.11
DBC	0.55+-0.121	0.5+-0.178	0.722+-0.081	0.714+-0.059
KNN	0.642+-0.195	0.474+-0.124	0.792+-0.077	0.703+-0.075
RF	0.753+-0.126	0.562+-0.128	0.864+-0.095	0.732+-0.102
Ridge	0.722+-0.113	0.604+-0.108	0.841+-0.074	0.805+-0.089
SVC	0.754+-0.106	0.606+-0.132	0.863+-0.067	0.782+-0.09

Table 8: Exemplary classification report for DBC in 5-fold, stratified cross-validation on the dataset by Xin et al. We observe low precision and recall for the classes PP and delta, where the respective inter- and intraclass distributions show high similarities.

	**Precision**	**Recall**	**F_1-Score**	**Support**
PP	0.16	0.66	0.26	85
alpha	0.95	0.73	0.83	886
beta	0.93	0.71	0.8	472
delta	0.04	0.08	0.05	49
**Accuracy**			0.7	1492
**Macro Average**	0.52	0.55	0.49	1492
**Weighted Average**	0.87	0.7	0.76	1492

6 Comparison of Conventional Machine Learning Methods and Deep Learning Approaches for the Baseline Classifier

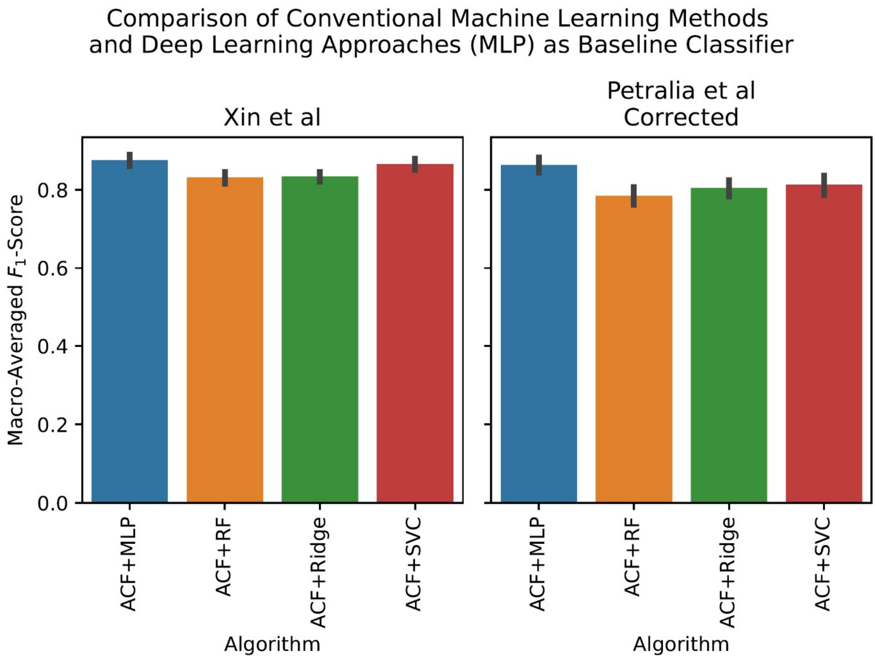

Figure 4: Comparison of conventional machine learning methods and a deep learning approach (Multi-layer perceptron – MLP) as baseline classifier for ACF. We considered an exemplary dataset for scRNA-seq and proteomics each (left and right respectively). The combination of ACF with the deep feed-forward neural network yields slightly higher macro-averaged F_1-scores than the three conventional machine learning methods (Random Forest – RF , Ridge-Classifier – Ridge and Support-Vector-Classifier – SVC).
